# Pregnancy outcomes in women with kidney transplant: Metaanalysis and systematic review

**DOI:** 10.1186/s12882-019-1213-5

**Published:** 2019-01-23

**Authors:** Silvi Shah, Renganathan Lalgudi Venkatesan, Ayank Gupta, Maitrik K. Sanghavi, Jeffrey Welge, Richard Johansen, Emily B. Kean, Taranpreet Kaur, Anu Gupta, Tiffany J. Grant, Prasoon Verma

**Affiliations:** 1 Division of Nephrology Kidney C.A.R.E. Program, University of Cincinnati, 231 Albert Sabin Way, MSB 6112, Cincinnati, OH 45267 USA; 2 Department of Environmental Health, University of Cincinnati, Cincinnati, OH USA; 3 Health Sciences Library, College of Medicine, University of Cincinnati, Cincinnati, OH USA; 4 Buffalo Medical Group, Buffalo, NY USA; 5 Division of Neonatology, Cincinnati Children’s Hospital and Medical Center, Cincinnati, OH USA

**Keywords:** Pregnancy, Kidney transplant, Maternal, Fetal, Outcomes

## Abstract

**Background:**

Reproductive function in women with end stage renal disease generally improves after kidney transplant. However, pregnancy remains challenging due to the risk of adverse clinical outcomes.

**Methods:**

We searched PubMed/MEDLINE, Elsevier EMBASE, Scopus, BIOSIS Previews, ISI Science Citation Index Expanded, and the Cochrane Central Register of Controlled Trials from date of inception through August 2017 for studies reporting pregnancy with kidney transplant.

**Results:**

Of 1343 unique studies, 87 met inclusion criteria, representing 6712 pregnancies in 4174 kidney transplant recipients. Mean maternal age was 29.6 ± 2.4 years. The live-birth rate was 72.9% (95% CI, 70.0–75.6). The rate of other pregnancy outcomes was as follows: induced abortions (12.4%; 95% CI, 10.4–14.7), miscarriages (15.4%; 95% CI, 13.8–17.2), stillbirths (5.1%; 95% CI, 4.0–6.5), ectopic pregnancies (2.4%; 95% CI, 1.5–3.7), preeclampsia (21.5%; 95% CI, 18.5–24.9), gestational diabetes (5.7%; 95% CI, 3.7–8.9), pregnancy induced hypertension (24.1%; 95% CI, 18.1–31.5), cesarean section (62.6, 95% CI 57.6–67.3), and preterm delivery was 43.1% (95% CI, 38.7–47.6). Mean gestational age was 34.9 weeks, and mean birth weight was 2470 g. The 2–3-year interval following kidney transplant had higher neonatal mortality, and lower rates of live births as compared to > 3 year, and < 2-year interval. The rate of spontaneous abortion was higher in women with mean maternal age < 25 years and > 35 years as compared to women aged 25–34 years.

**Conclusion:**

Although the outcome of live births is favorable, the risks of maternal and fetal complications are high in kidney transplant recipients and should be considered in patient counseling and clinical decision making.

**Electronic supplementary material:**

The online version of this article (10.1186/s12882-019-1213-5) contains supplementary material, which is available to authorized users.

## Background

Women with end stage renal disease have impaired fertility due to disruption of hypothalamic gonadal axis. Pregnancy is therefore rare in women on dialysis with very low incidence of conception ranging from 0.9 to 7% [1]. Since there is rapid restoration of fertility, in some cases, within 6 months following transplantation, kidney transplantation offers the best hope to women with end-stage renal disease who wish to become pregnant [2].

Pregnancy in a kidney transplant recipient continues to remain challenging due to risk of adverse maternal complications of preeclampsia and hypertension, and risk of adverse fetal outcomes of premature birth, low birth weight, and small for gestational age infants [3]. Additionally, there is risk of side effects from immunosuppressive medication, and risk of deterioration of allograft function [4]. Therefore, preconception counseling, family planning and contraception are pertinent parts of the transplant counseling process.

Data on clinical outcomes of pregnancy in kidney transplant recipients is limited from case reports, single-center studies, and voluntary registries. The usefulness of the voluntary registries is further limited due to underreporting and incomplete data capture [5–8]. To the best of our knowledge, no comprehensive metanalysis on post-kidney transplant pregnancy outcomes has been performed in the recent years [9]. Since kidney transplant is common in women of child bearing age and most of the data on outcomes of pregnancy comes from these retrospective studies, our metaanalysis is both timely and important. The comprehensive analysis of various worldwide registries, single-center studies, and case series will provide generalizable inferences about post-kidney transplant pregnancy outcomes, and help guide the pregnancy in kidney transplant recipients. The primary goal of this study was to perform a meta-analysis to systematically identify all studies of pregnancy-related outcomes in kidney transplant recipients from all around the world, and estimate pooled incidences of pregnancy outcomes, maternal complications, and fetal complications. The secondary goals were to examine the impact of pregnancy on the kidney allograft loss, allograft rejection, identify ideal maternal age of conception, and determine ideal time of conception between kidney transplant and pregnancy.

## Methods

### Data sources and searches

**Fig. 1:**
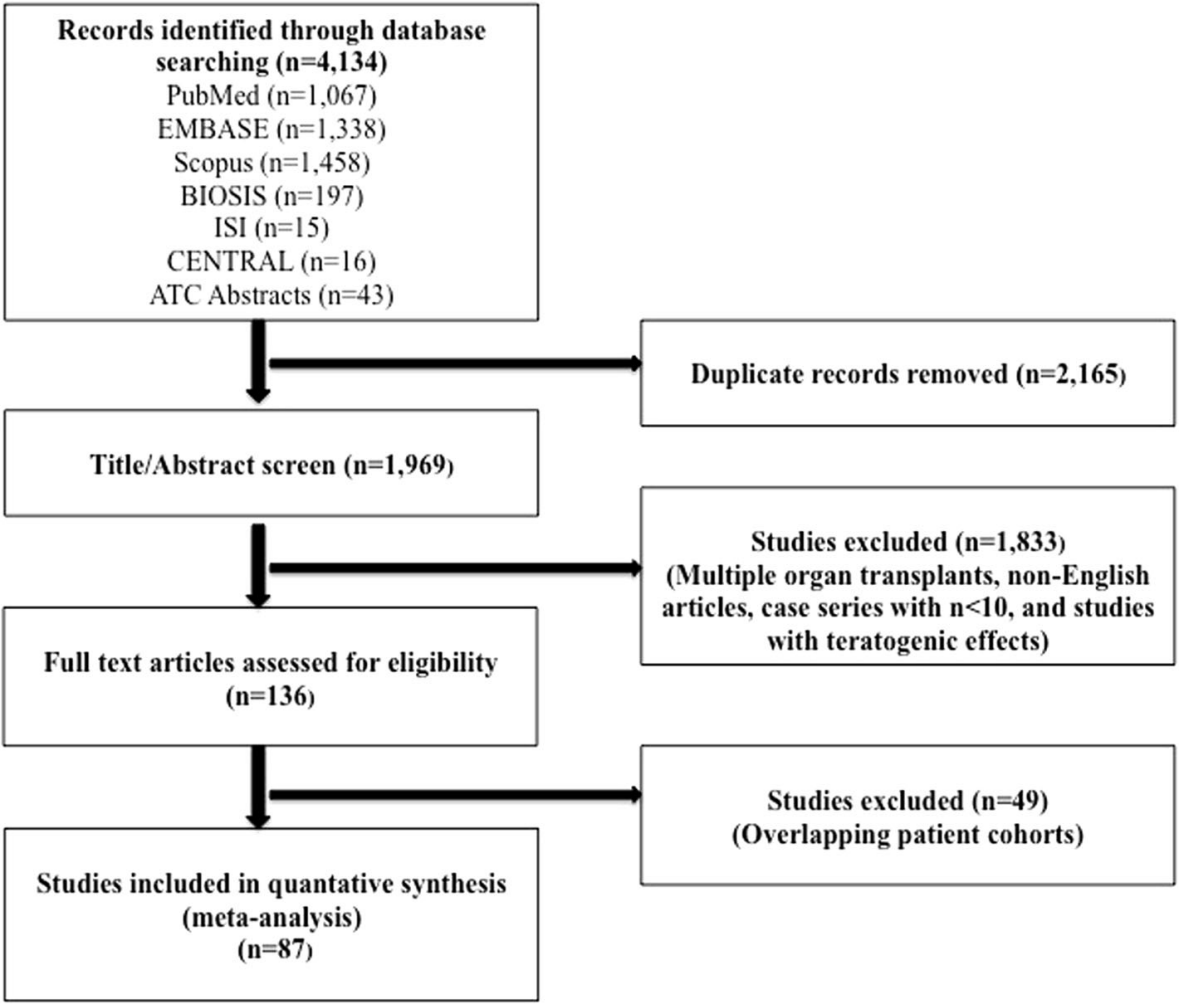
Study cohort showing the selection of studies reporting outcomes of pregnancies in women with kidney transplant

We performed a systematic review and meta-analyses reported according to PRISMA guidelines for studies exploring incidence and outcomes of pregnancy in women with kidney transplant (Fig. 1). We searched PubMed/MEDLINE, Elsevier EMBASE, Scopus, BIOSIS Previews, ISI Science Citation Index Expanded, and the Cochrane Central Register of Controlled Trials (CENTRAL) from their earliest date of inception through 8/31/2017, and abstracts from the annual American Transplant Congresses from 1/1/2013 through 8/31/2017. A health sciences librarian (E.K.) developed database-specific search strategies including a combination of subject headings (MeSH or Emtree) and keywords. The following key search terms were used in strategies specific to each database and organization: pregnancy complications, pregnancy outcome, maternal outcome, fetal outcome, birth outcome, kidney transplant, or renal transplant. A reproducible PubMed search strategy is provided in Additional file 1.

### Study selection

We considered observational studies (prospective cohort, retrospective cohort, and cross-sectional), case series, and case reports (with *n* > 10 pregnancies) that explored the pregnancy, maternal, and fetal outcomes among women ≥18 years, and who received a kidney transplant. Studies of patients with multiple organ transplants, studies that analyzed the teratogenic effects of mycophenolate or sirolimus, and non–English language studies were excluded. Titles and abstracts of all identified citations were screened independently by two reviewers (S.S. and T.G.), who discarded studies that did not meet all inclusion criteria. The same reviewers independently screened the abstracts of all eligible studies. If eligibility was indeterminable from the abstract, the study was included in the full-text screen. All disagreements were adjudicated by the principal investigator (S.S).

### Data extraction, quality assessment, and outcomes

Data extraction was carried out independently by three data extraction team members (A.G, L.R. and M.S.) using standard data extraction forms. Data elements were then rechecked for accuracy by all the three data extraction team members. When more than one publication of a similar patient population existed with more than 25% overlap, publication with higher number of pregnancy events and the most complete details was included. Disagreements in data extraction and quality assessment were resolved in consultation with an arbitrator (T.G.) and primary investigator (S.S.). For each included study, the following data was extracted: country of location, years of data collection, number of kidney transplant recipients, number of pregnancies, mean maternal age, mean interval between kidney transplant and pregnancy, pregnancy outcomes (number of live births, miscarriage, induced abortion, still birth and ectopic pregnancies), maternal outcomes (number of women with preeclampsia, pregnancy induced hypertension, and gestational diabetes mellitus, and number of cesarean sections), fetal outcomes (number of pre-term births, mean gestational age, mean gestational weight, and number of neonatal deaths); and graft outcomes (number of acute rejection during pregnancy, graft failure post pregnancy, mean serum creatinine pre and post pregnancy). To maintain consistency across extracted data, the number of pregnancies was used as a denominator for the outcomes of live births, miscarriages, induced abortions, stillbirths, neonatal deaths, preeclampsia, pregnancy induced hypertension, and gestational diabetes mellitus. The number of live births was used as the denominator for the outcome of preterm deliveries, and cesarean section. Preterm was defined as babies born alive before 37 weeks gestation.

### Data synthesis and analysis

Patients characteristics were reported as frequencies. The pregnancy incidence was reported for women per 1000 live births. For each study, estimates were expressed as prevalence and 95% confidence intervals (CI). Prevalence estimates from individual studies were pooled using a random-effects model. Heterogeneity across included studies was analyzed formally using Cochran Q (heterogeneity 2) and I2 statistics. For binary outcomes, the DerSimonian-Laird method was used, and for continuous outcomes, a weighted average methodology was used to calculate the pooled estimates and 95% CI. Two-sample test of proportions was used to compare the pooled incidence for each analysis to the most recent United States (US) general population incidence. [10–14] We determined the associations of maternal age, the interval between kidney transplant, and the pregnancy outcomes. Additonally, we performed a subgroup analysis for the pregnancy, maternal and fetal outcomes for studies published from 2000 to 2017. Analyses were performed using MS Excel and Comprehensive Meta-analysis packages in R software.

## Results

Among the 4134 citations that were retrieved, 136 full-text articles were reviewed and 87 were selected to be included in the final study cohort (Fig. 1). Three studies were from Africa, 31 from Asia, 31 from Europe, 10 from North America, 4 from Oceania, and 8 from South America **(**Table 1**)**. Overall, there were 6712 pregnancies in 4174 kidney transplant recipients. Mean maternal age was 29.6 ± 2.4 years and mean interval between kidney transplant and pregnancy was 3.7 years.

**Table 1:** Studies included in the metaanalysis

	Reference, Year Published	Study Years	Country	Recipients	Pregnancies
1	Devresse et al., 2017 [32]	1994–2010	Belgium	32	57
2	Yuksel et al., 2017 [33]	2009–2016	Turkey	25	na
3	Ajaimy et al., 2016 [34]	2009–2014	USA	11	11
4	Candido et al., 2016 [35]	2004–2014	Portugal	36	53
5	Cristelli et al., 2016 [36]	2004–2014	Brazil	36	53
6	El Houssni et al., 2016 [37]	na	Saudi Arabia	12	21
7	Lima et al., 2016 [38]	2004–2014	Brazil	36	53
8	Majak et al., 2016 [39]	1969–2013	Norway	na	119
9	Mishra et al., 2016 [40]	2004–2014	India	16	na
10	Orihuela et al., 2016 [41]	1986–2014	Uruguay	32	40
11	Piccoli et al., 2016 [42]	1978–2013	Italy	na	189
12	Saliem et al., 2016 [43]	2006–2011	Canada	na	264
13	Santos et al., 2016 [44]	2010–2014	Portugal	8	8
14	Sarween et al., 2016 [45]	2001–2015	UK	387	569
15	Stoumpos et al., 2016 [46]	1973–2013	UK	89	138
16	Yoshikawa et al., 2016 [47]	na	Japan	49	65
17	Aktrurk et al., 2015 [48]	2004–2014	Turkey	12	16
18	Arab et al., 2015 [49]	2003–2010	Canada	na	375
19	Erman et al., 2015 [50]	1987–2011	Turkey	43	43
20	Yeon et al., 2015 [51]	1995–2015	Korea	84	119
21	Debska – Slizien et al., 2014 [52]	1980–2012	Poland	17	22
22	Farr et al., 2014 [53]	1999–2013	Austria	12	12
23	Hebral et al., 2014 [54]	1969–2011	France	46	61
24	You et al., 2014 [55]	1995–2013	Korea	29	41
25	Blume et al., 2013 [56]	1988–2010	Germany	34	53
26	Guella et al., 2013 [57]	1992–2008	Saudi Arabia	15	33
27	Pietrzak et al., 2013 [58]	2001–2012	Poland	34	40
28	Rachdi et al., 2013 [59]	2003–2013	Tunisia	12	17
29	Ribeiro et al., 2013 [60]	1995–2007	Brazil	22	31
30	Rocha et al., 2013 [61]	1983–2009	Portugal	24	25
31	Wyld et al., 2013 [62]	1971–2010	Australia	447	692
32	Kennedy et al., 2012 [63]	na	Ireland	18	29
33	Neyatani et al., 2012 [64]	1975–2011	Japan	22	34
34	Van Buren et al., 2012 [65]	1971–2010	Netherlands	30	42
35	Celik et al., 2011 [66]	1998–2008	Turkey	24	31
36	Gerlei et al., 2011 [67]	1974–2010	Hungary	23	27
37	Lopez et al., 2011 [68]	1986–2010	Spain	20	24
38	Xu et al., 2011 [69]	1989–2008	China	25	38
39	Gorgulu et al., 2010 [70]	1983–2008	Turkey	19	22
40	Areia et al., 2009 [71]	1989–2007	Portugal	28	34
41	Gill et al., 2009 [20]	1990–2003	USA	483	530
42	Levidiotis et al., 2009 [8]	1966–2005	Australia	381	577
43	Rizvi et al., 2009 [72]	1985–2008	Pakistan	45	72
44	Sharma et al., 2009 [73]	1988–2006	Oman	42	82
45	Al Duraihimh et al., 2008 [74]	1996–2006	Middle East	140	234
46	Alfi A Yet al, 2008 [75]	1989–2005	Saudi Arabia	12	20
47	Cruz Lemini et al., 2007 [28]	1990–2005	Mexico	60	75
48	Oliveira et al., 2007 [76]	2001–2005	Brazil	52	52
49	Sibanda et al., 2007 [77]	1994–2001	UK	176	193
50	Yassaee et al., 2007 [78]	1996–2001	Iran	74	95
51	Kurata et al., 2006 [79]	1984–2003	Japan	42	53
52	Rahamimov et al., 2006 [80]	1983–1998	Israel	39	69
53	Galdo et al., 2005 [81]	1982–2002	Chile	30	37
54	Garcia - Donaire et al., 2005 [82]	1997–2004	Spain	16	19
55	Ghanem et al., 2005 [83]	1989–2004	Egypt	41	67
56	Pour-Reza-Gholi et al., 2005 [84]	1984–2004	Iran	60	74
57	Yildirim et al., 2005 [85]	1998–2005	Turkey	17	20
58	Keitel et al., 2004 [86]	1977–2001	Brazil	41	44
59	Pezeshki et al., 2004 [87]	1991–1998	Iran	18	20
60	Hooi et al., 2003 [88]	1984–2001	Malaysia	46	72
61	Queipo et al., 2003 [89]	1980–2000	Spain	29	40
62	Thompson et al., 2003 [90]	1976–2001	UK	24	48
63	Sgro et a,l 2002 [91]	1988–1998	Canada	26	44
64	Tan et al., 2002 [92]	1986–2000	Singapore	25	42
65	Park et al., 2001 [93]	na - 2000	South Korea	36	47
66	Kuvacic et al., 2000 [94]	1986–1996	Croatia	15	23
67	Little et al., 2000 [27]	1985–1998	Ireland	19	29
68	Moon et al., 2000 [95]	na - 1998	Korea	36	48
69	Ventura et al., 2000 [96]	1983–1999	Portugal	15	15
70	Arsan et al., 1997 [97]	na	France	20	33
71	Rahbar et al., 1997 [98]	1985–1993	Iran	13	14
72	Rieu et al., 1997 [99]	1970–1995	France	22	33
73	Al Hassani et al., 1995 [100]	1985–1993	Oman	25	44
74	Sabagh et al., 1995 [101]	1984–1994	Saudi Arabia	33	52
75	Saber et al., 1995 [102]	1968–1992	Brazil	19	25
76	Wong et al., 1995 [103]	1972–1992	New Zealand	9	16
77	Hadi et al., 1986 [104]	1969–1992	South Korea	11	13
78	Talaat et al., 1994 [105]	1977–1992	Sweden	19	25
79	Pahl et al., 1993 [106]	1969–1990	USA	21	32
80	Muirhead et al., 1992 [107]	1977–1988	UK	22	22
81	Brown et al., 1991 [108]	1965–1989	Ireland	14	27
82	Sturgiss et al., 1991 [109]	1967–1987	UK	17	22
83	O’ Connell et al., 1989 [110]	1974–1986	Australia	11	18
84	Ha et al., 1994 [111]	1970–1982	USA	13	17
85	Marushak et al., 1986 [112]	1972–1983	Denmark	20	24
86	O’ Donnell et al., 1985 [113]	1971–1984	South Africa	21	38
87	Waltzer et al., 1980 [114]	na	USA	12	15

*na* not available

### Pregnancy outcomes

**Fig. 2:**
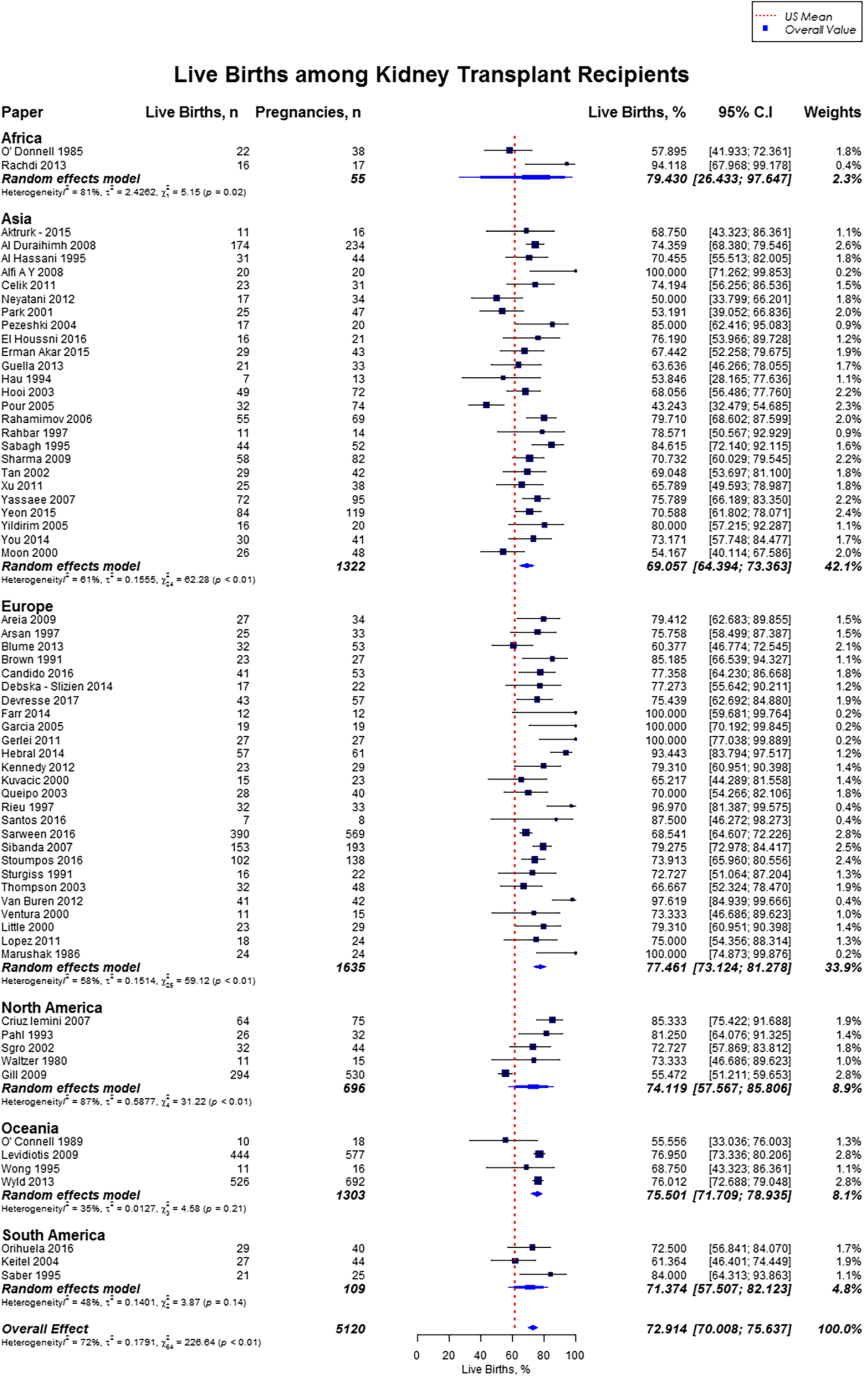
Forest Plot showing outcome of live births among kidney transplant recipients overall, and across different geographical regions

**Fig. 3:**
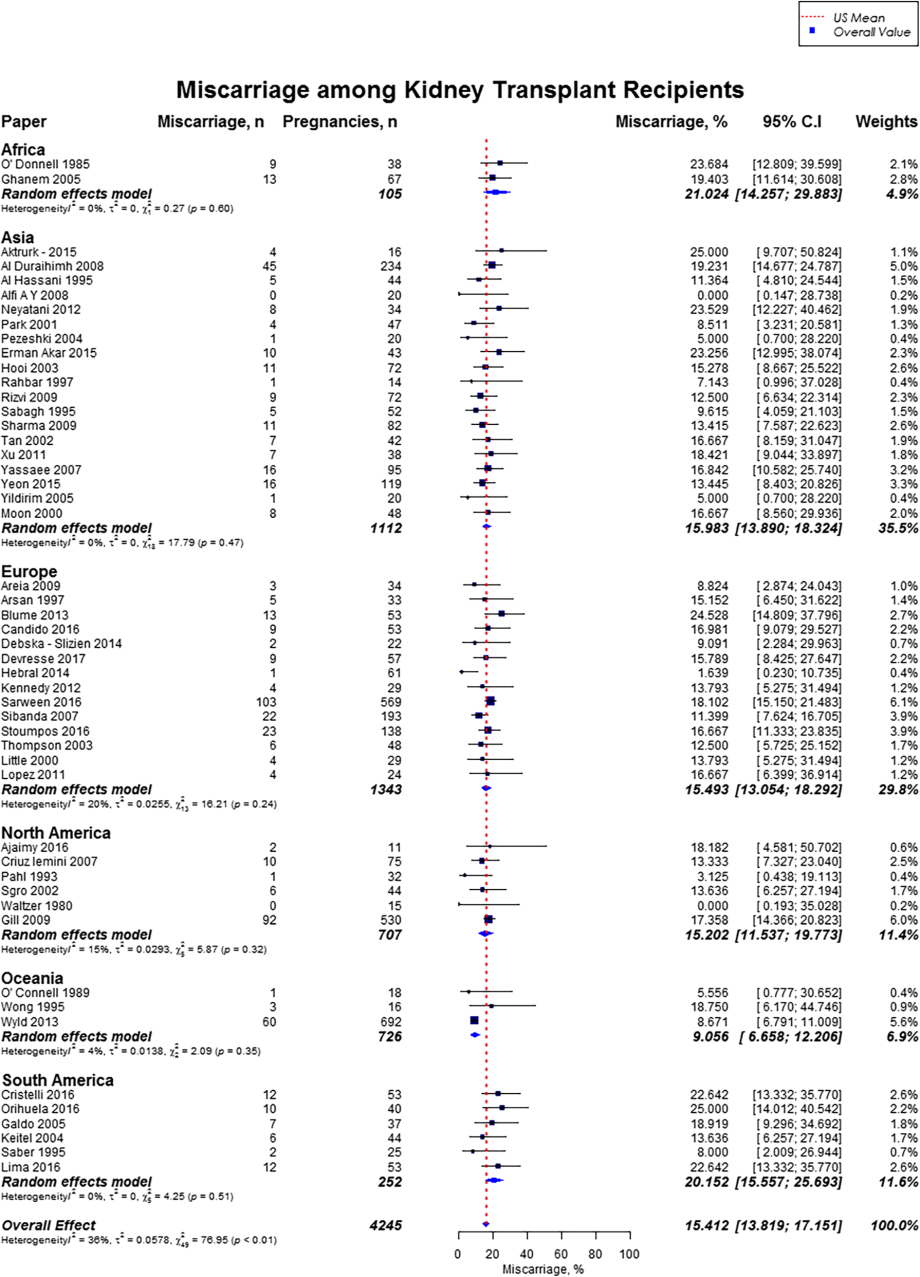
Forest Plot showing outcome of miscarriages among kidney transplant recipients overall, and across different geographical regions

**Fig. 4:**
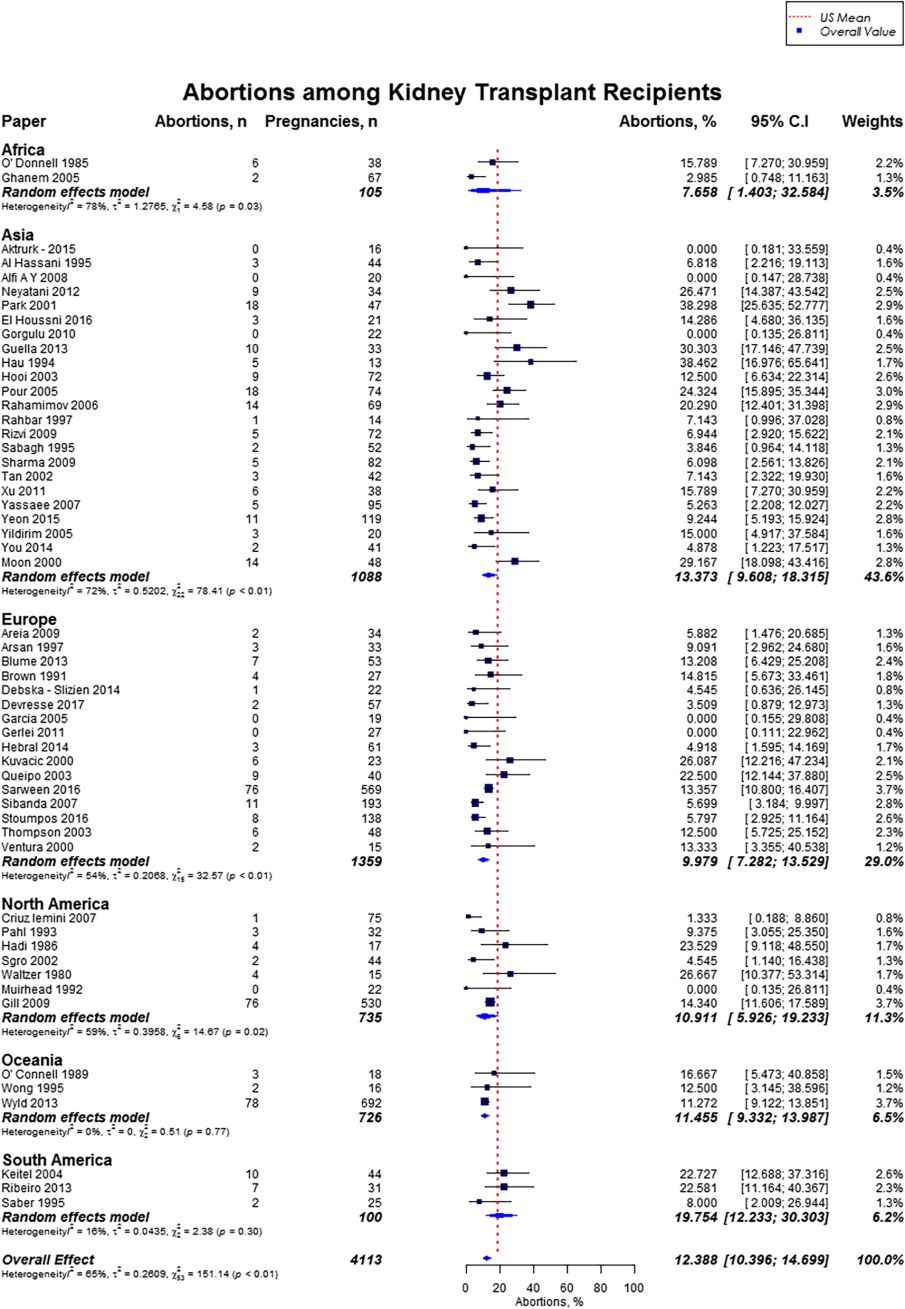
Forest Plot showing outcome of induced abortions among kidney transplant recipients overall, and across different geographical regions

**Fig. 5:**
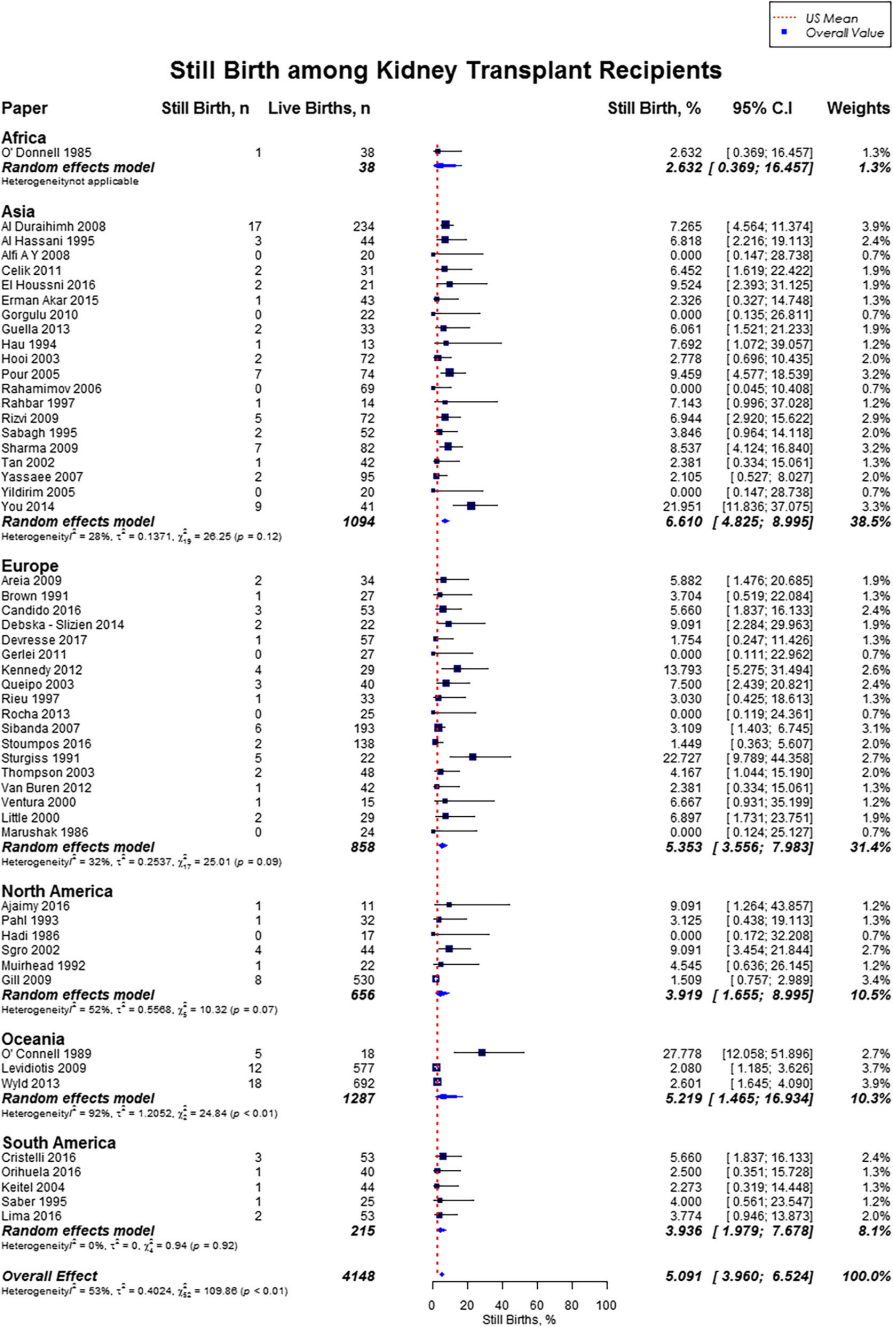
Forest Plot showing outcome of still births among kidney transplant recipients overall, and across different geographical regions

**Fig. 6:**
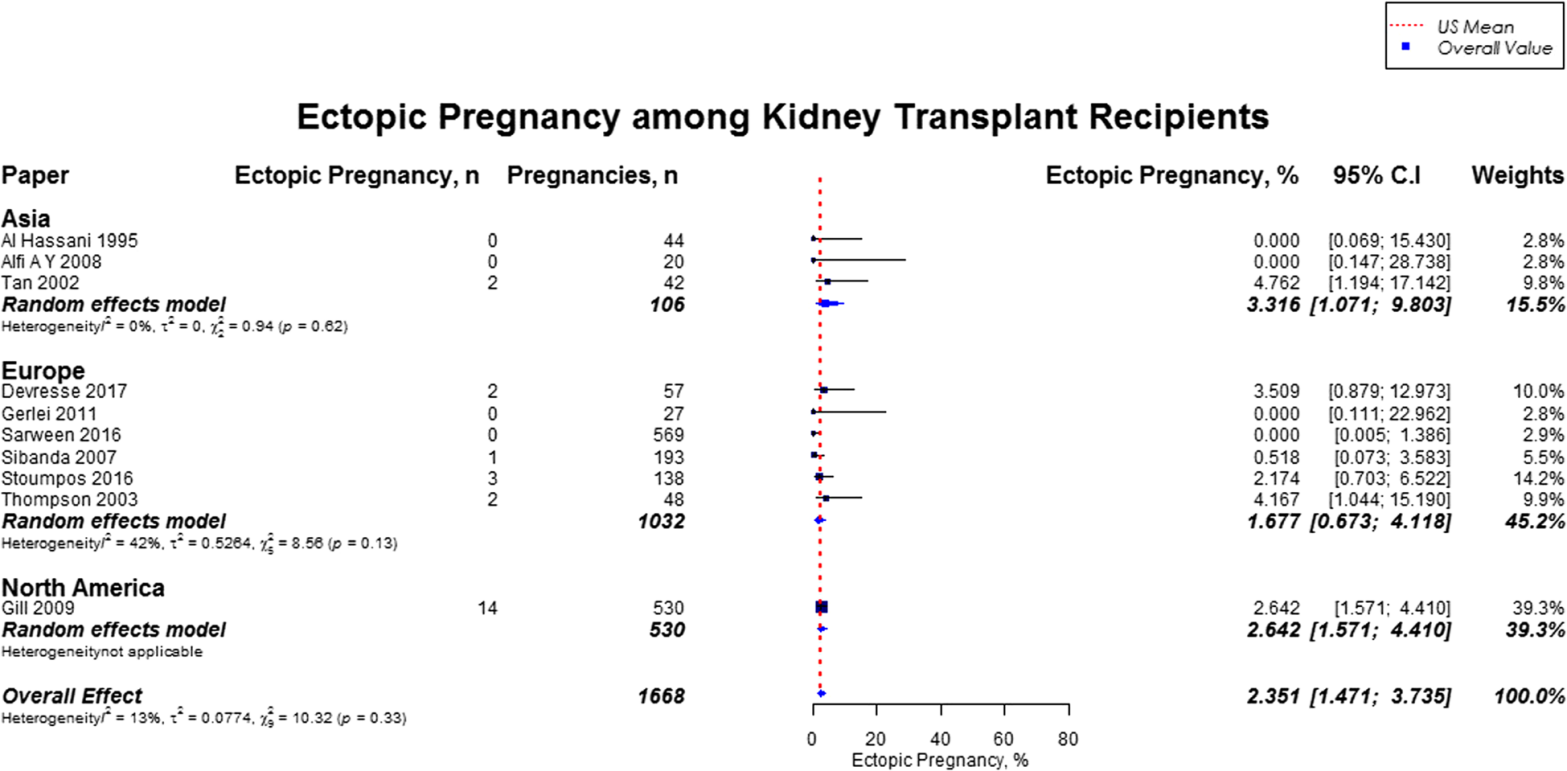
Forest Plot showing outcome of ectopic pregnancies among kidney transplant recipients overall, and across different geographical regions

Live birth rate was 72.9% (95% CI, 70.0–75.6), miscarriages rate was 15.4% (95% CI, 13.8–17.2), induced abortions rate was 12.4% (95% CI, 10.4–14.7), stillbirths rate was 5.1% (95% CI, 4.0–6.5) and rate of ectopic pregnancies was 2.4% (95% CI, 1.5–3.7). In our study cohort of kidney transplant recipients, live birth rates were higher as compared to the US general population (72.9% vs. 62%) and favorable across all geographic regions (Fig. 2) [10, 11]. Overall, miscarriage rate was slightly lower than that of the US general population (15.4% vs. 17.1%), but higher across Africa (21.0%; 95% CI, 14.3–29.9), and South America (20.2%; 95% CI, 15.6–25.7) (Fig. 3) [13]. Induced abortion rate was also lower than the US general population (12.4% vs. 18.6%) [13]. The rate of induced abortion was highest in South America (19.8%; 95% CI, 12.2–30.3), followed by Asia (13.3%; 95% CI, 9.6–18.3), Oceania (11.5%; 95% CI, 9.3–14.0), North America (10.9%; 95% CI, 5.9–19.2), Europe (10.0%; 95% CI, 7.3–13.5), and Africa (7.7, 95% CI, 1.4–32.6) **(**Fig. 4). Overall, stillbirth rate was higher than the US general population (5.1% vs. 0.6%) [14]. Worldwide, stillbirth rate was highest in Asia (6.6, 95% CI, 4.8–9.0%), and lowest in Africa (2.6, 95% CI; 0.4–16.5) **(**Fig. 5). The rate of ectopic pregnancy was slightly higher than the US general population (2.4% vs. 1.4%), with highest rate in Asia (3.3, 95% CI; 1.1–9.8) **(**Fig. 6) [15]. The results from the subgroup analyses (2000–2017) for pregnancy outcomes were consistent with the current findings (Additional file 2).

### Maternal outcomes

**Fig. 7:**
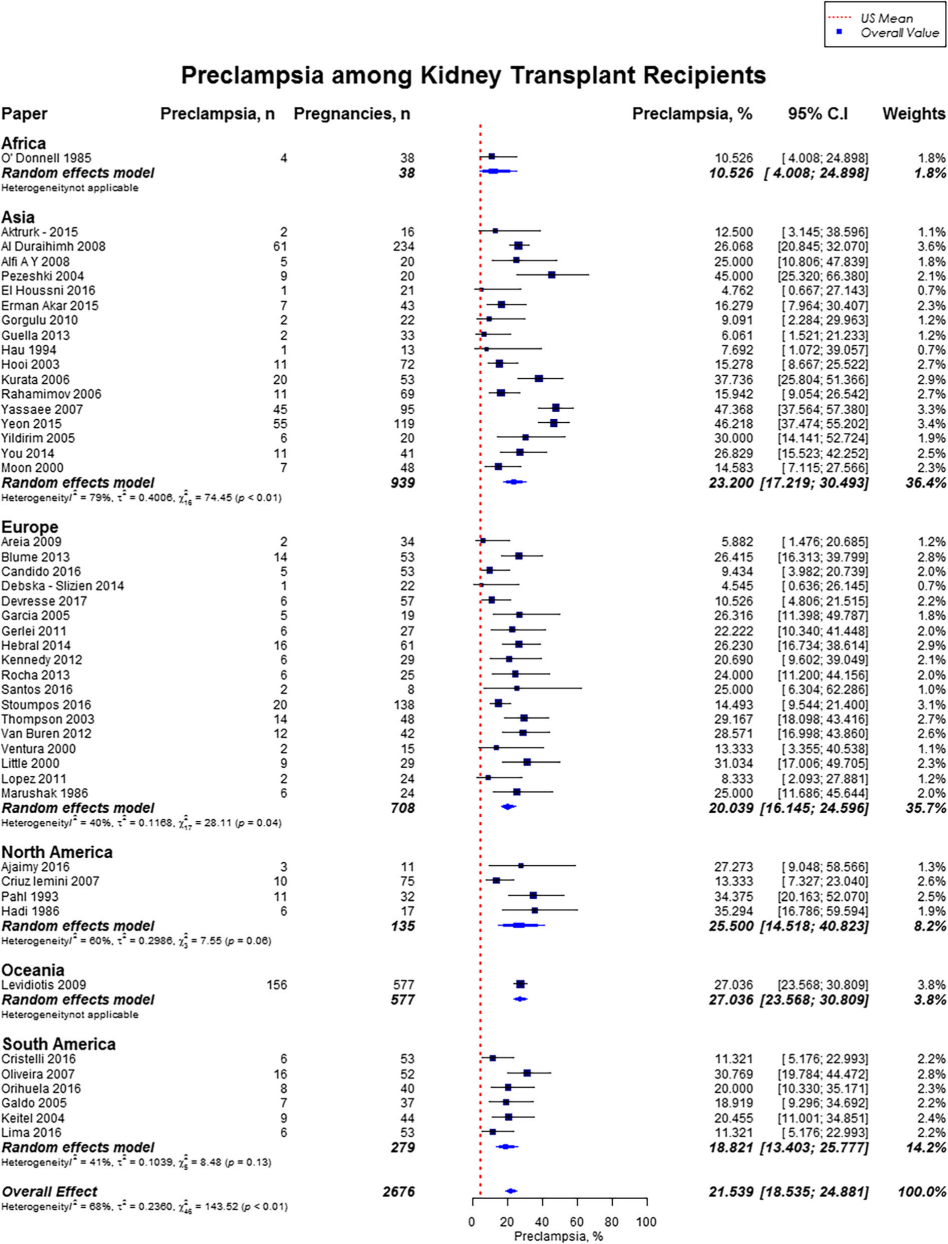
Forest Plot showing outcome of preeclampsia among kidney transplant recipients overall, and across different geographical regions

**Fig. 8:**
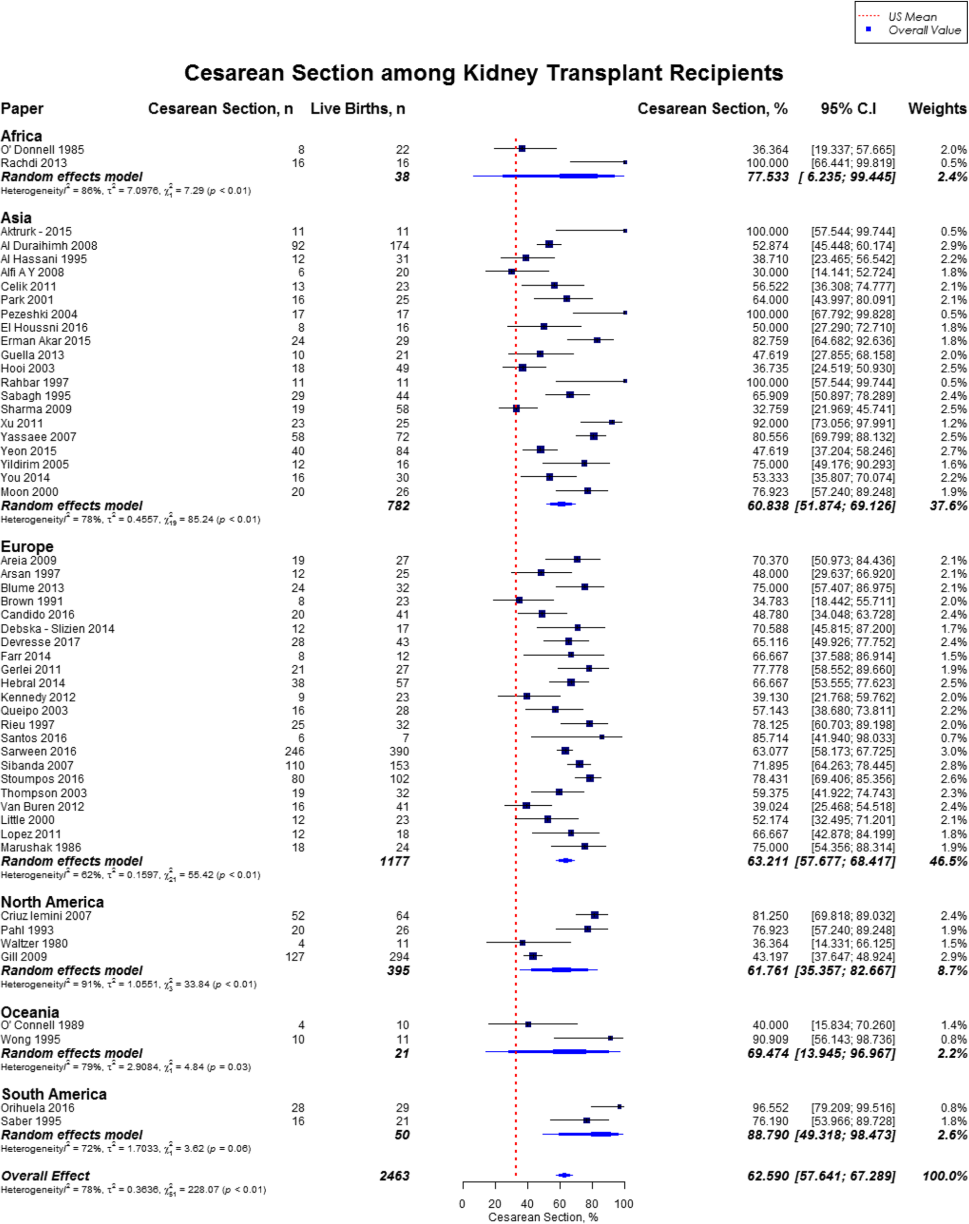
Forest Plot showing outcome of cesarean section among kidney transplant recipients overall, and across different geographical regions

**Fig. 9:**
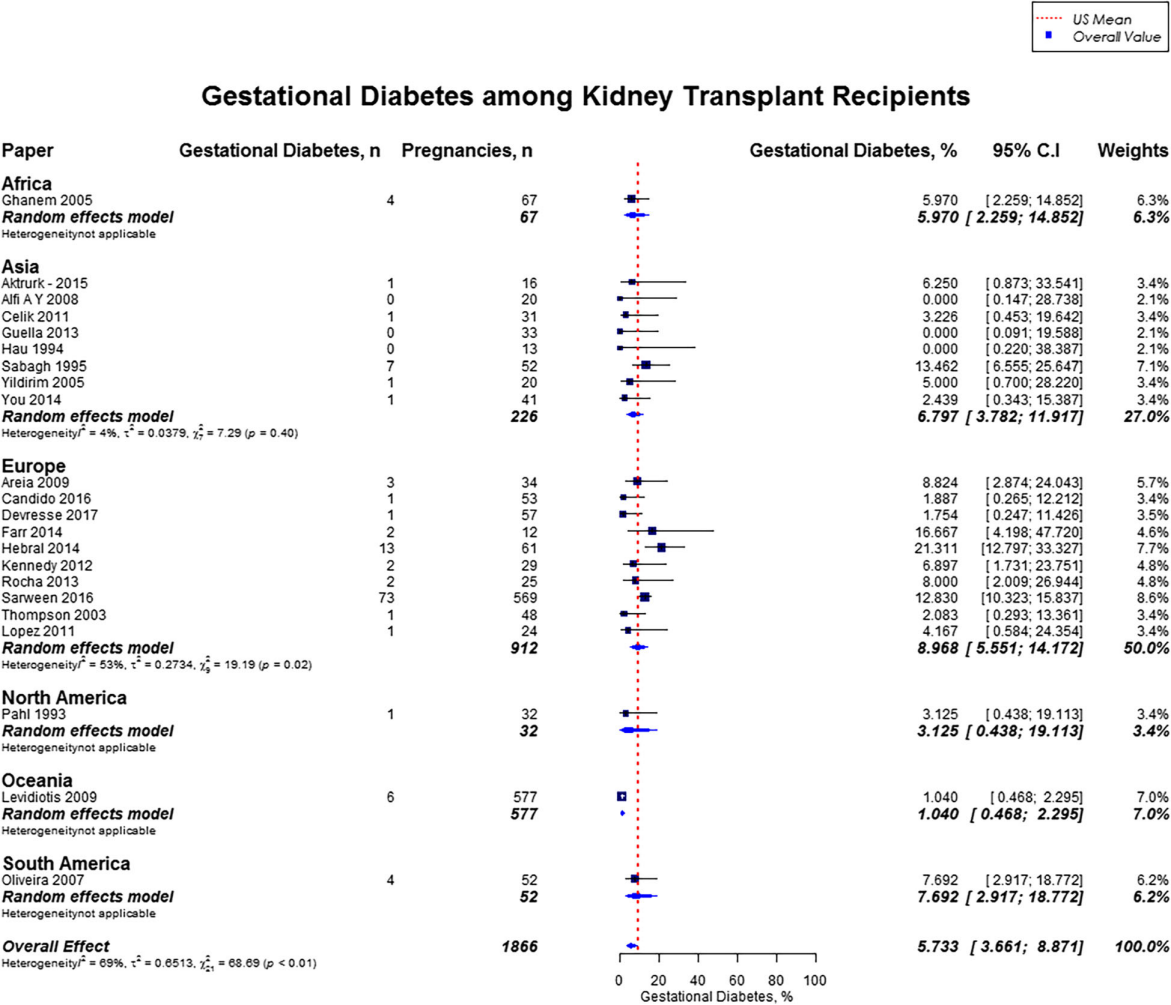
Forest Plot showing outcome of gestational diabetes among kidney transplant recipients overall, and across different geographical regions

**Fig. 10:**
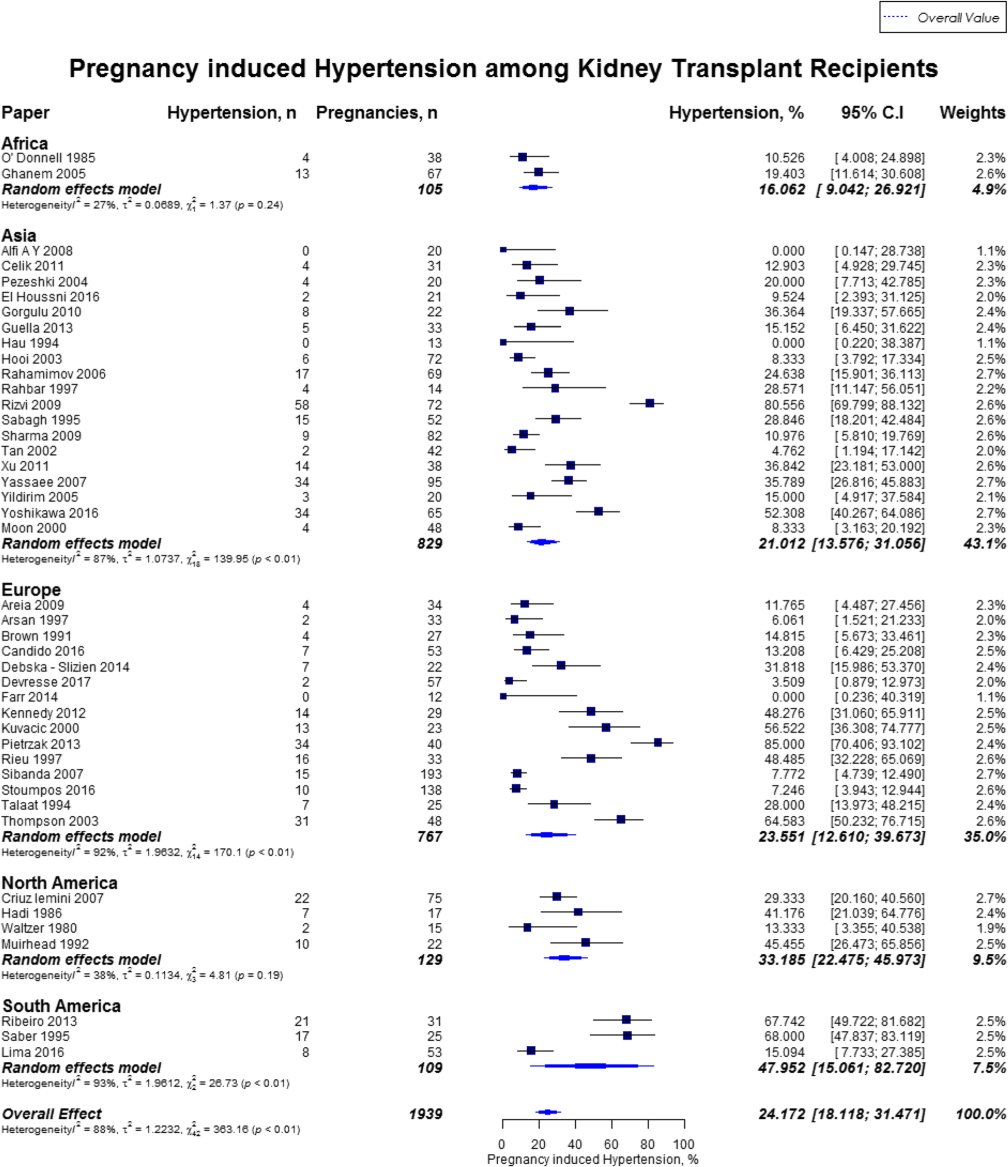
Forest Plot showing outcome of pregnancy induced hypertension among kidney transplant recipients overall, and across different geographical regions

Overall, rates of preeclampsia was 21.5% (95% CI, 18.5–24.9; US mean, 3.8%), cesarean section was 62.6% (95% CI, 57.6–67.3; US mean, 31.9%), gestational diabetes was 5.7% (95% CI, 3.7–8.9; US mean, 9.2%), and pregnancy induced hypertension was 24.1% (95% CI, 18.1–31.5). [12, 16] Preeclampsia rate was highest in Oceania (27.0%; 95% CI, 23.6–30.8), followed by North America (25.5%; 95% CI, 14.5–40.8), and lowest in Africa (10.5%; 95% CI, 4.0–24.9%) (Fig. 7). Cesarean section rate was highest in South America (88.8%; 95% CI, 49.3–98.5), followed by Africa (77.5%; 95% CI, 6.3–99.4) (Fig. 8). Worldwide, Oceania had the lowest rates of gestational diabetes (1.0%; 95% CI, 0.5–2.3%) (Fig. 9). With regards to pregnancy induced hypertension, highest rate was reported in South America (48.0, 95% CI, 15.1–82.7), while lowest rate was in Africa (16.1, 95% CI, 9–26.9) (Fig. 10). The results from the subgroup analyses (2000–2017) for maternal outcomes were consistent with the current findings (Additional file 2).

### Fetal outcomes

**Fig. 11:**
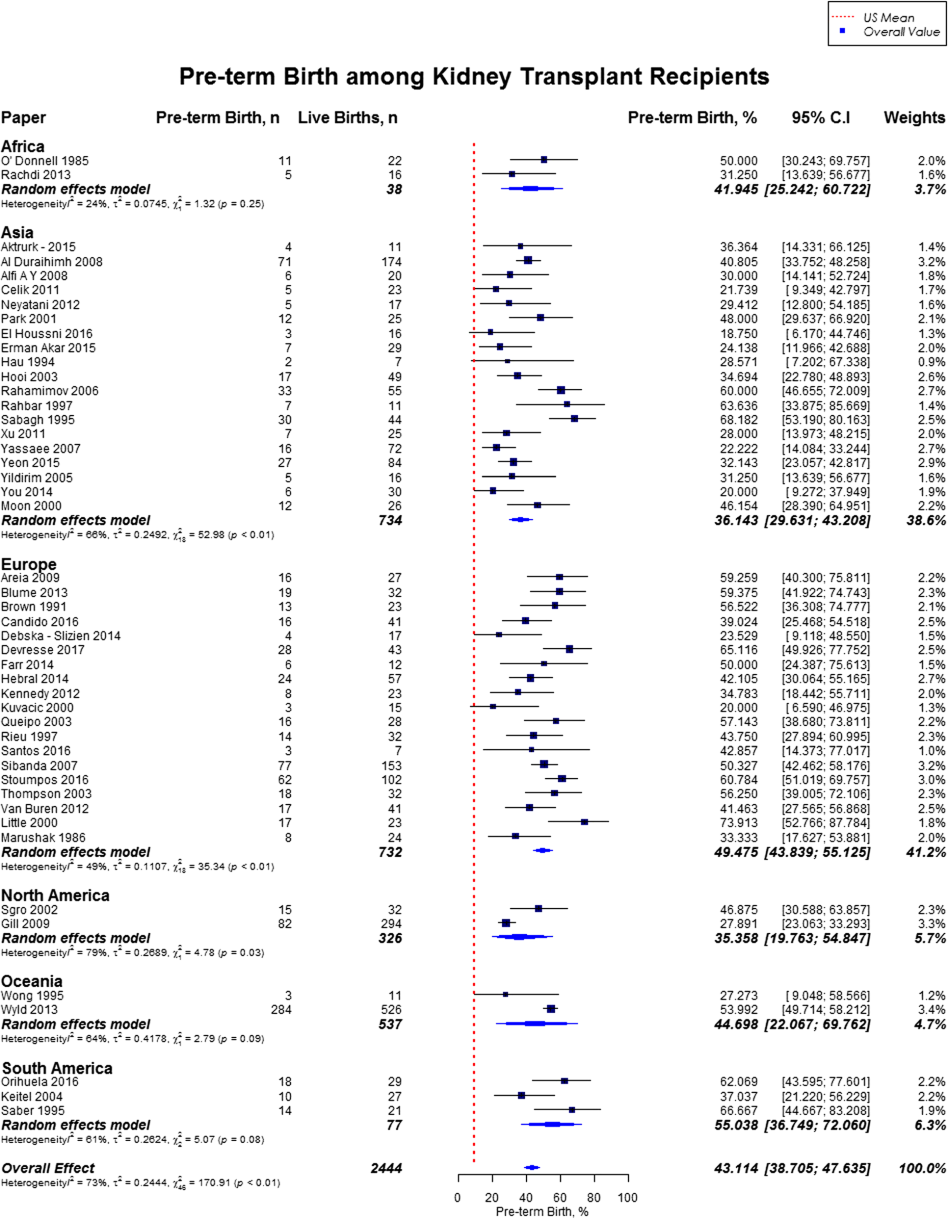
Forest Plot showing outcome of preterm births among kidney transplant recipients overall, and across different geographical regions

**Fig. 12:**
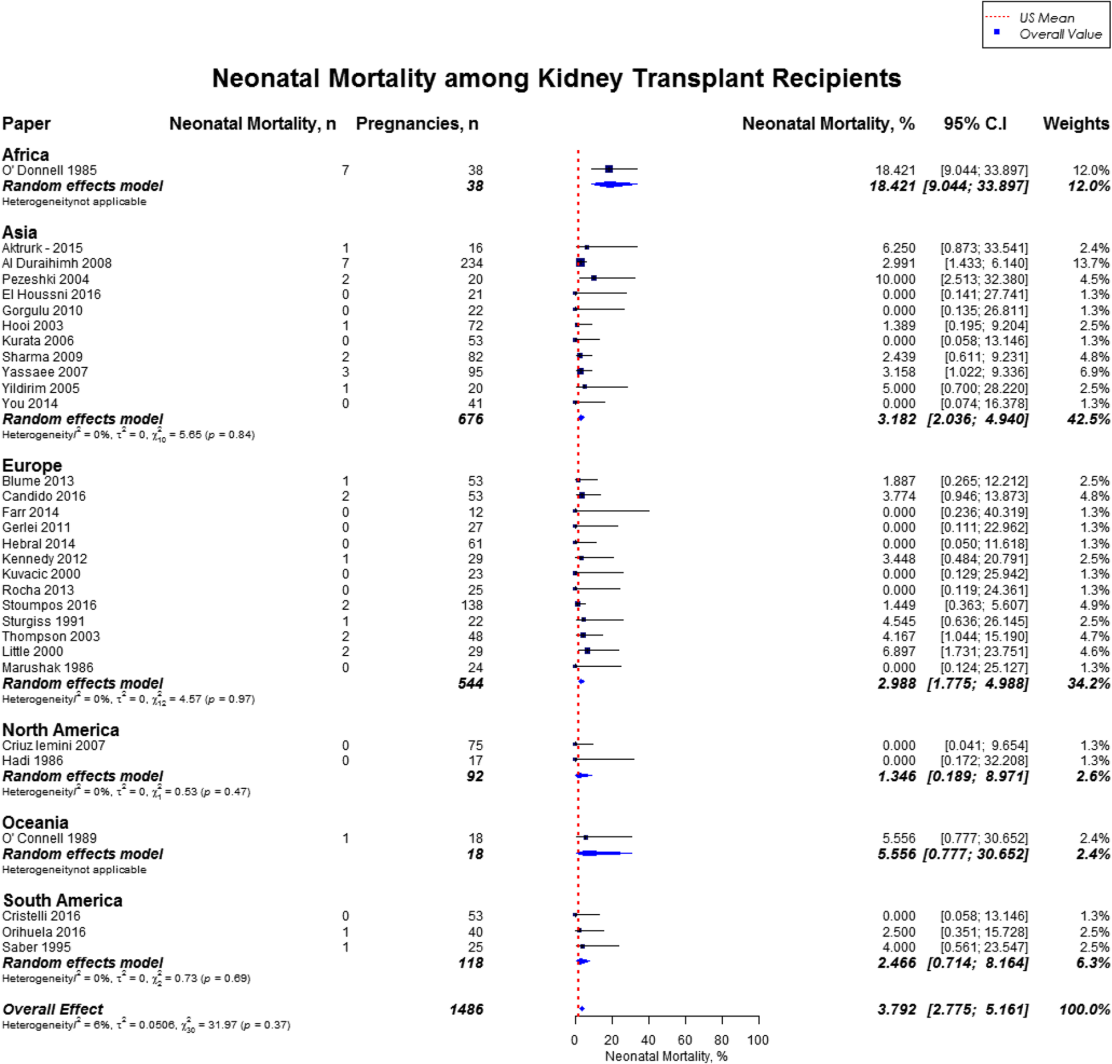
Forest Plot showing outcome of neonatal mortality among kidney transplant recipients overall, and across different geographical regions

Overall, rate of preterm birth was 43.1% (95% CI, 38.7–47.6) defined by babies born alive before 37 weeks of gestation, and neonatal mortality was 3.8% (95% CI, 2.8–5.2). Rates of preterm birth was highest in South America (55.0%), and lowest in North America (35.4%) (Fig. 11). The mean gestational age for newborns was 34.9 weeks (US mean, 38.7 weeks) and the mean birth weight was 2470 g (US mean, 3389 g). [12, 17] Neonatal mortality was high across all geographical regions as compared to the US mean (3.8% vs. 0.4%), with highest rate in Africa (18.4%; 95% CI, 9.1–33.9) and lowest rate in North America (1.3, 95% CI, 0.2–8.9) **(**Fig. 12**)** [18]. The results from the subgroup analyses (2000–2017) for fetal outcomes were consistent with the present findings except for neonatal mortality which was slighly lower in the subgroup analysis (2.9% vs. 3.8%) (Additional file 2).

### Graft outcomes

**Fig. 13:**
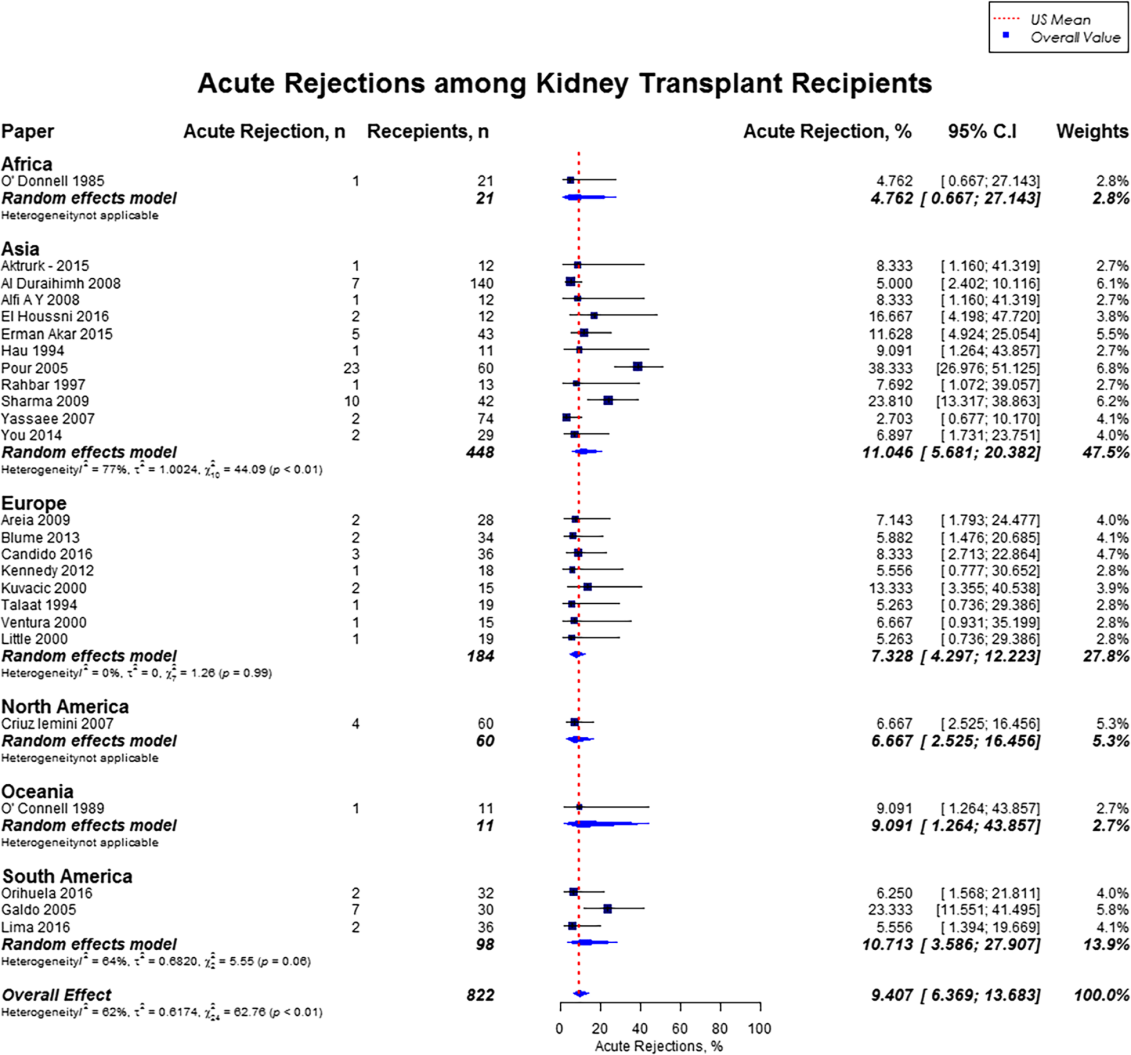
Forest Plot showing rates of acute rejection among kidney transplant recipients overall, and across different geographical regions

The overall acute rejection rate during pregnancy among 822 kidney transplant recipients was 9.4% (95% CI, 6.4–13.7), which was comparable to the US mean of 9.1%. [19] Rates of acute renal allograft rejection were highest in Asia (11.0%), followed by South America (10.7%), Oceania (9.1%), Europe (7.3%), North America (6.7%), and Africa (4.8%) (Fig. 13). With regards to graft failure, there was large variability in the follow up period ranging from 1 year to 14 years. However among 489 recipients in 12 studies where two-year post pregnancy graft loss was reported, there were 32 cases of graft loss (9.2%). The change in preconception creatinine and post-pregnancy creatinine, was statistically significant (1.23 ± 0.16 mg/dl vs. 1.37 ± 0.27 mg/dl, *p* = 0.007).

### Time of conception

Outcomes were also stratified by interval of < 2 years, 2–3 years, and > 3 years between pregnancy and kidney transplant (Table 2). Adverse pregnancy outcomes of induced abortion rates and neonatal deaths were highest in the 2–3 year interval following kidney transplant as compared to < 2 year interval and > 3 year interval (16% vs. 11% vs. 10, and 9% vs. 3% vs. 4% respectively). Cesarean section rate and live birth rate were also less favorable in this interval of 2–3 years than > 3 year, and < 2 year interval (68% vs. 75% vs. 74, and 73% vs. 65% vs. 42% respectively). Maternal complication of preeclampsia was higher in the 2–3 interval, and > 3 year interval than < 2 year interval (24% vs. 23% vs. 13%). Spontaneous abortion rates were highest in > 3 year interval followed by 2–3 interval, and < 2 interval (16% vs. 14% vs. 10%).

**Table 2:** Pregnancy-related outcomes stratified by study mean interval between transplant and pregnancy

	< 2 years	> 2–3 years	> 3 years
Number of papers	4	15	44
Number of pregnancies	149	835	3182
Mean maternal age (year)	28.3	29.4	29.1
Pregnancy Outcomes			
Live birth	73.8%	68.3%	75.4%
Induced abortion	10.7%	16.1%	10.2%
Spontaneous abortion	10.3%	14.0%	16.3%
Still birth	6.7%	5.1%	3.7%
Neonatal deaths	3.4%	9.3%	3.7%
Cesarean section	41.8%	72.7%	64.5%
Maternal Outcomes			
Preeclampsia	13.2%	24.3%	22.8%
Pregnancy induced hypertension	12.1%	30.8%	23.0%
Gestational diabetes	0.0%	8.8%	7.2%
Fetal Outcomes			
Pre-term delivery	41.9%	41.6%	45.4%
Mean gestation time (weeks)	36.1	34.5	34.9
Birth weight (grams)	2349.00	2533.21	2460.79
Graft Outcomes			
Acute rejection	8.1%	5.1%	3.0%
Graft loss	16.7%	14.6%	6.3%

### Maternal age for conception

We further stratified the pregnancy, maternal, fetal, and graft outcomes by maternal age categories (Table 3). Lower live birth rate was observed in women with maternal age 29–34 years than those < 29 years (74% vs. 76%). Rates of spontaneous abortion were highest in women < 25 years and > 35 years followed by women with maternal age 25–34 years (20% vs. 18% vs. 11%). Preeclampsia rates were higher in women with maternal age > 35 years (27%) and 29–34 years (26%) followed by < 25 years (17%) and 25–29 years (14%).

**Table 3:** Pregnancy-related outcomes stratified by study mean maternal age

Study mean maternal age (years)				
	< 25	25–29	30–34	≥35
Number of papers	3	22	35	1
Number of pregnancies	103	723	3474	11
Mean maternal age (year)	23.3	27.4	30.2	36.0
Pregnancy Outcomes				
Live birth	75.8%	75.8%	73.9%	na
Induced abortion	14.0%	11.3%	11.0%	na
Spontaneous abortion	19.8%	16.0%	13.3%	18.2%
Still birth	2.9%	5.3%	3.6%	9.1%
Neonatal deaths	na	5.4%	3.0%	na
Cesarean section	48.0%	68.3%	63.6%	na
Maternal Outcomes				
Preeclampsia	17.1%	13.7%	26.5%	27.3%
Pregnancy induced hypertension	16.5%	25.2%	23.4%	na
Gestational diabetes	na	5.8%	7.0%	na
Fetal Outcomes				
Pre-term delivery	na	46.4%	47.4%	na
Mean gestation time (weeks)	35.5	35.5	34.6	na
Birth weight (grams)	2460.0	2607.7	2456.9	na
Graft Outcomes				
Acute rejection	3.8%	3.3%	5.8%	na
Graft loss	na	12.1%	10.4%	27.3%

*na not available

## Discussion

The results of our meta-analysis show that although majority of pregnancies in women after kidney transplant result in live birth, both maternal and fetal adverse events are common. Rates of preeclampsia, still birth, and cesarean section were significantly higher than in the general population. In the cohort considered for the analysis, a quarter of women had serious pregnancy complications, defined as at least one of preterm delivery, first or second trimester loss, stillbirth, or neonatal death. Additionally, rates of preterm delivery, still births, and neonatal mortality were higher as compared with the US recent national data.

The live birth rates in women after kidney transplant were higher than in the general population (73% vs. 62%) and this trend was consistent throughout the globe [10]. Our study confirms the findings from The National Transplant Pregnancy Registry from the United States that reported a live birth rate of 71–76% [7]. Similarly, meta-analysis done by Deshpande et al. examined pregnancy outcomes of 4706 pregnancies in women with kidney transplant and reported a live birth rate of 73.5% [9]. The higher live birth rate, although appears encouraging, may reflect a reporting bias or a selection bias in which relatively healthy women decided to pursue pregnancy, and subsequently received better medical support by multiple specialties. It is also important to consider that there are inconsistencies in definition of live birth rate used in various studies, for example live birth rate was defined as per 1000 female transplant recipients in some studies, whereas per 1000 pregnancies in transplant recipients in others [7, 20]. Live birth rate in general population (comparison group) is defined by Centers for Disease Control as live births per 1000 population [10]. Additionally, it remains unclear how the multiple gestation pregnancy outcomes were evaluated in these studies. Contrary to the above findings of successful pregnancies, a US health utilization study found a much lower live birth rate of 55% in kidney transplant recipients. They attributed this finding of low live birth rate to underestimation of fetal loss [20]. Davison et al. estimated that just under 40% of conceptions do not go beyond the first trimester, but of those that do, greater than 90% end successfully [21]. Another explanation of high live birth rate in our study could be the exclusion of studies that reported pregnancy outcomes with teratogenic immunosuppressive medications of mycophenolate and sirolimus.

Our study highlights the significantly higher risk of maternal and fetal complications in women with kidney transplants. About a quarter of women developed preeclampsia, and the rates of preeclampsia were almost six fold higher as compared to the general US population (21.5% vs. 3.8%) [16]. Vannevel et al. in an international multicenter retrospective cohort of 52 women who underwent kidney transplantation reported preeclampsia rate of as high as 38%, and chronic hypertension rate of 27% [22]. Hypertension is common in kidney transplant recipients prior to conception with a reported incidence of 52 to 69% [1]. Several factors can contribute to the onset of hypertension after renal transplantation, including but not limited to the type of immunosuppressive therapy (calcineurin inhibitors and corticosteroids), allograft function, donor type, obesity, alcohol, smoking, and presence of a native kidney (increased production of renin) [23]. Diagnosis of superimposed preeclampsia can be difficult in kidney transplant patients due to higher frequency of preexisting hypertension and proteinuria [1, 24].

We found significant differences in rates of gestational diabetes mellitus between various geographical location, for example rates were as high as 8.9% in Europe and as low as 1% in Oceania. Although, the increased rate of gestational diabetes in kidney transplant patients can be well explained by the use of immunosuppressive medications like steroids and calcineurin inhibitors, the striking differences between rates of gestational diabetes according to geographic location also highlights the importance of predisposition to diabetes due to ethnicity. Unfortunately, it was not possible to evaluate the differences in immunosuppressive medications as usually they are individualized to the needs of the patients and transplant center protocol [1, 25].

Rates of stillbirth and neonatal mortality were significantly higher in our study as compared to the general population. While prior studies have not reported higher rates of neonatal mortality and stillbirths in kidney transplant recipients, the current study finding is highly significant. Possible reasons could be prematurity, preeclampsia or presence of other risk factors like hypertension, proteinuria, and serum creatinine of 1.5 mg/dl or higher [26–28]. While it was not possible to determine the exact cause for stillbirth or neonatal mortality, this study finding is critical for counselling of women of child bearing age contemplating pregnancy. In our study, the rate of cesarean section was higher than two folds as compared to general population in United States, and varied from 60 to 77% across different geographical locations. Bramham et al. reported that more than three quarters of the deliveries in kidney transplant recipients were by cesarean section, but only 3% were performed for the indication of renal transplant [3]. Vaginal delivery should not be impaired in kidney transplant patients, as the pelvic allograft does not obstruct the birth canal in most patients [1]. This exceptionally higher rates of cesarean sections in kidney transplant recipients can be attributed to fetal and maternal complications, but warrants further study. There was a high rate of premature births in the transplant population in the present study and close to half of the live births were premature deliveries. Prior studies have showed a preterm birth rate of 40–60% in kidney transplant recipients [9, 29]. Fetal complications, suspected renal compromise or preeclampsia are some of the common indications of early iatrogenic delivery. Interestingly, only quarter of preterm deliveries in renal transplant recipients are induced [3, 7].

The optimal time to conception after renal transplant continues to remain an area of contention. The ideal time of conception in women with renal transplant is between 1 and 2 years after transplantation according to guidelines by American Society of Transplantation, whereas European best practice guidelines recommend delaying pregnancy for a period of 2 years after transplantation [30, 31]. In our study, live birth rate was lowest and neonatal deaths were highest in the 2–3 year interval following kidney transplant. Maternal complication of cesarean section and preeclampsia were higher in the 2–3 and > 3 year interval. In contrast, Deshpande et al. reported both the highest maternal complications of preeclampsia, cesarean section, and gestational diabetes, and least favorable delivery outcome of preterm births in the < 2 year interval as compared to > 2 year interval between kidney transplant and pregnancy [9]. However their analysis was limited by inclusion of only 3 studies in the < 2 interval following kidney transplant. Overall, fetal outcomes in < 2 year interval seem most favorable in our study but merits further investigation due to limitation of the retrospective study design, small numbers, and possible reporting bias associated with data from voluntary registries.

A significant strength of our study is that it involves a large number of pregnant renal transplant recepients from all around the globe, thus providing us with information about pregnancy outcomes for a heterogenous population. Additionally, we have analyzed region specific outcomes and identified outcomes which may require intensive management pertaining to that region. This will help in making future region specific guidelines for follow up and management of pregnancy in kidney transplant recpients. The following limitations should be considered when interpreting the findings of our study. We examined pregnancy outcomes over several decades in the present study. While it is expected for the outcomes to change due to improvement in obstetric care in kidney transplant recipients over the course of time, subgroup analysis for studies from 2000 to 2017 showed consistent results. There were inconsistencies in the definition of live birth rate amongst different studies that may have affected the results. Reporting bias may have affected the miscarriage rate. We were unable to account for differences in socioeconomics, and healthcare conditions among the different geographic regions. Due to lack of individual patient data, we were not able to assess pregnancy outcomes in relation to immunosuppression regimens.

## Conclusions

This meta analysis of pregnancy outcomes in 6712 pregnancies in 4174 kidney transplant recipients with data spread over different decades from all over the world shows favorable outcomes with live birth rates exceeding that in the recent national population. Majority of patients preserve their graft. However, pregnancy after renal transplant confers significant risk in terms of maternal and fetal adverse events, including increased rates of preeclampsia, gestational diabetes, cesarean section rates, and pregnancy induced hypertension. The risk of prematurity and low birth rate are also high. Areas which need to be studied in the future include type of immunosuppression and its correlation with specific pregnancy outcomes; and evaluation of risk factors associated with specific maternal and fetal adverse events. The definitions used in evaluating these outcomes also need to be standardized. The results of this study can help the health care providers with appropriate counseling and individualized management of this high risk population.

## Additional files

Additional file 1: Reproducible search strategy. (DOCX 149 kb)

Additional file 2: Subgroup analysis of various pregnancy outcomes in kidney transplant recipients for studies published from 2000 to 2017. (DOCX 16 kb)

## Abbreviations

CI: Confidence interval
US: United States
