# Supplementary material for: Pregnancy outcomes in women with kidney transplant: Metaanalysis and systematic review
Source: BMC Nephrol. 2019 Jan 23;20:24. doi: 10.1186/s12882-019-1213-5 (PMC6345071; doi:10.1186/s12882-019-1213-5)
Supplement: Supplementary file 1 — Reproducible search strategy. (DOCX 149 kb) [file 12882_2019_1213_MOESM1_ESM.docx]

Supplement 1.

Full PubMed Search String (Basis for translation to all databases):

(((pregnancy complications[mesh:noexp] OR pregnancy complication*[tiab] OR Abortion, Spontaneous[mesh:noexp] OR Pregnancy Outcome[mesh:noexp] OR pregnancy outcome*[tiab] OR Abortion, Habitual[mesh:noexp] OR Abortion, Septic[mesh:noexp] OR Diabetes, Gestational[mesh:noexp] OR gestational diabetes[tiab] OR pregnancy diabetes[tiab] OR pregnancy induced diabetes[tiab] OR pregnancy-induced diabetes[tiab] OR maternal diabetes mellitus[tiab] OR maternal death[mesh:noexp] OR maternal death*[tiab] OR Hypertension, Pregnancy-Induced[mesh:noexp] OR pregnancy induced hypertension[tiab] OR pregnancy-induced hypertension[tiab] OR gestational hypertension[tiab] OR Eclampsia[mesh:noexp] OR Eclampsia[tiab] OR Pre-Eclampsia[mesh:noexp] OR pre-eclamp*[tiab] OR pre eclamp*[tiab] OR preeclamp*[tiab] OR preeclamp*[ot] OR pre-eclamp*[ot] OR preeclamptic toxemia*[tiab] OR pre-eclamptic toxemia*[tiab] OR pre eclamptic toxemia*[tiab] OR toxemia[tiab] OR Maternal Mortality[mesh] OR maternal mortalit*[tiab] OR maternal outcome*[tiab] OR maternal outcome*[ot] OR fetal death[mesh:noexp] OR fetal death*[tiab] OR fetal mortalit*[tiab] OR infant death*[tiab] OR infant mortalit*[tiab] OR Stillbirth[mesh:noexp] OR Stillbirth*[tiab] OR Still birth*[tiab] OR Still-birth*[tiab] OR Fetal Diseases[mesh:noexp] OR Fetal Disease*[tiab] OR Erythroblastosis, Fetal[mesh:noexp] OR fetal erythroblastosis[tiab] OR Fetal Growth Retardation[mesh] OR Fetal Growth Retardation[tiab] OR Perinatal Death[mesh] OR perinatal death*[tiab] OR peri-natal death*[tiab] OR Infant, Low Birth Weight[mesh:noexp] OR Infant, Small for Gestational Age[mesh:noexp] OR Infant, Very Low Birth Weight[mesh:noexp] OR Infant, Extremely Low Birth Weight[mesh] OR birth weight*[tiab] OR Small for Gestational Age infant[tiab] OR Small for gestational age[tiab] OR Intrauterine growth restriction*[tiab] OR IUGR[tiab] OR Perinatal Mortality[mesh] OR perinatal mortalit*[tiab] OR peri-natal mortalit*[tiab] OR Obstetric Labor Complications[mesh:noexp] OR Obstetric complication*[tiab] OR Uterine Complication*[tiab] OR labor complication*[tiab] OR labour complication*[tiab] OR obstetric outcome*[tiab] OR labor outcome*[tiab] OR labour outcome*[tiab] OR Fetal Membranes, Premature Rupture[mesh:noexp] OR Fetal Membranes Premature Rupture[tiab] OR Obstetric Labor, Premature[mesh:noexp] OR Premature Obstetric Labor[tiab] OR Premature Labor[tiab] OR Premature Labour[tiab] OR Early Labor[tiab] OR Early Labour[tiab] OR Premature Birth[mesh] OR Premature Birth*[tiab] OR Premature infant*[tiab] OR Prematurity[tiab] OR Preterm birth*[tiab] OR Pre-term birth*[tiab] OR Preterm deliver*[tiab] OR Pregnancy Complications, Hematologic[mesh] OR Pregnancy Complications, Infectious[mesh] OR Pregnancy Complications, Cardiovascular[mesh] OR Pregnancy Complications, Hematologic[mesh] OR Pregnancy Complications, Infectious[mesh] OR Puerperal Infection[mesh] OR Puerperal Infection*[tiab] OR Pregnancy Complications, Neoplastic[mesh] OR Trophoblastic Neoplasms[mesh:noexp] OR Trophoblastic Neoplasm*[tiab] OR Choriocarcinoma[mesh] OR Choriocarcinoma[tiab] OR Pregnancy in Diabetics[mesh] OR Pregnancy in Diabet*[tiab] OR Prenatal Exposure Delayed Effects[mesh] OR Prenatal Exposure Delayed Effect*[tiab] OR Puerperal Disorders[mesh] OR Puerperal Disorder*[tiab] OR Infant, Premature, Diseases[mesh:noexp] OR premature infant disease*[tiab] OR birth outcome*[tiab] OR birth outcome*[ot] OR abortion*[tiab] OR miscarriage*[tiab] OR delivery outcome*[tiab] OR pregnancy toxemia*[tiab] OR maternal hypertension[tiab] OR neonatal mortalit*[tiab] OR neonatal death*[tiab] OR newborn death*[tiab] OR new-born death*[tiab] OR new born death*[tiab] OR newborn mortalit*[tiab] OR new-born mortalit*[tiab] OR new born mortalit*[tiab] OR Proteinuria Edema Hypertension Gestosis[tiab] OR Post-neonatal Mortalit*[tiab] OR Post neonatal Mortalit*[tiab]))) AND ((kidney transplantation[mesh] OR Kidney Transplant*[tiab] OR Kidney Transplant*[ot] OR Kidney transplant recipient*[tiab] OR Kidney Recipient*[tiab] OR Renal Transplant*[tiab] OR Renal Transplant*[ot] OR Renal Transplant Recipient*[tiab] OR post-renal transplant pregnanc*[tiab] OR postrenal transplant pregnanc*[tiab] OR Kidney Allograft*[tiab] OR Kidney Allograft Transplantation*[tiab] OR Kidney Allotransplantation*[tiab] OR Kidney Graft*[tiab] OR Renal Graft*[tiab]))
