# Supplementary material for: Pregnancy outcomes in women with kidney transplant: Metaanalysis and systematic review
Source: BMC Nephrol. 2019 Jan 23;20:24. doi: 10.1186/s12882-019-1213-5 (PMC6345071; doi:10.1186/s12882-019-1213-5)
Supplement: Supplementary file 2 — Subgroup analysis of various pregnancy outcomes in kidney transplant recipients for studies published from 2000 to 2017. (DOCX 16 kb) [file 12882_2019_1213_MOESM2_ESM.docx]

|  |  | 95% Confidence Interval |
| --- | --- | --- |
| Number of papers | 69 |  |
| Number of pregnancies | 6242 |  |
| Mean maternal age (years)* | 29.8 ± 2.3 |  |
| **Pregnancy Outcomes** |  |  |
| Live birth | 72.4 % | 69.2-75.4 |
| Induced abortion | 12.1% | 9.9-14.8 |
| Miscarriages | 15.8 % | 14.0-17.6 |
| Still birth | 4.7 % | 3.6-6.2 |
| Neonatal mortality | 2.9% | 2.1-4.1 |
| Cesarean section | 63.4 % | 57.8-68.6 |
| Ectopic pregnancies | 2.4 % | 1.4-3.9 |
| **Maternal Outcomes** |  |  |
| Preeclampsia | 21.3 % | 18.2-24.9 |
| Pregnancy induced hypertension | 23.3% | 16.2-32.3 |
| Gestational diabetes | 5.4 % | 3.3-8.9 |
| **Fetal Outcomes** |  |  |
| Preterm delivery | 41.6 % | 36.9-46.6 |
| Mean gestation time (weeks)* | 34.7 ± 2.25 |  |
| Mean birth weight (grams)* | 2473.7 ± 251.9 |  |
| **Graft Outcomes** |  |  |
| Acute rejection | 9.8% | 6.3-14.9 |

Supplement 2.

*Reported in mean ± standard deviation. Remaining outcomes reported in rates in percentage.
